# The Making of Leukemia

**DOI:** 10.3390/ijms19051494

**Published:** 2018-05-17

**Authors:** Inés González-Herrero, Guillermo Rodríguez-Hernández, Andrea Luengas-Martínez, Marta Isidro-Hernández, Rafael Jiménez, Maria Begoña García-Cenador, Francisco Javier García-Criado, Isidro Sánchez-García, Carolina Vicente-Dueñas

**Affiliations:** 1 Experimental Therapeutics and Translational Oncology Program, Instituto de Biología Molecular y Celular del Cáncer, CSIC/Universidad de Salamanca, Campus M. de Unamuno s/n, 37007 Salamanca, Spain; ighe@usal.es (I.G.-H.); guillermorh@usal.es (G.R.-H.); andrealuengas@usal.es (A.L.-M.); martaisidroh@gmail.com (M.I.-H.); 2 Institute of Biomedical Research of Salamanca (IBSAL), 37007 Salamanca, Spain; rajim@usal.es (R.J.); mbgc@usal.es (M.B.G.-C.); 3 Departamento de Fisiología y Farmacología, Universidad de Salamanca, Edificio Departamental, Campus M. de Unamuno s/n, 37007 Salamanca, Spain; 4 Departamento de Cirugía, Universidad de Salamanca, 37007 Salamanca, Spain

**Keywords:** leukemia, oncogenes, reprogramming, stem cells, cancer therapy, leukemia stem cell, mouse model

## Abstract

Due to the clonal nature of human leukemia evolution, all leukemic cells carry the same leukemia-initiating genetic lesions, independently of the intrinsic tumoral cellular heterogeneity. However, the latest findings have shown that the mode of action of oncogenes is not homogeneous throughout the developmental history of leukemia. Studies on different types of hematopoietic tumors have shown that the contribution of oncogenes to leukemia is mainly mediated through the epigenetic reprogramming of the leukemia-initiating target cell. This driving of cancer by a malignant epigenetic stem cell rewiring is, however, not exclusive of the hematopoietic system, but rather represents a common tumoral mechanism that is also at work in epithelial tumors. Tumoral epigenetic reprogramming is therefore a new type of interaction between genes and their target cells, in which the action of the oncogene modifies the epigenome to prime leukemia development by establishing a new pathological tumoral cellular identity. This reprogramming may remain latent until it is triggered by either endogenous or environmental stimuli. This new view on the making of leukemia not only reveals a novel function for oncogenes, but also provides evidence for a previously unconsidered model of leukemogenesis, in which the programming of the leukemia cellular identity has already occurred at the level of stem cells, therefore showing a role for oncogenes in the timing of leukemia initiation.

## 1. The Making of Leukemia: The Concept of Epigenetic Reprogramming

In spite of the enormous amount of data that we have gathered in the last four decades about the biology of tumor cells, our capacity to control the development of the disease is still very limited [1,2]. We still do not know how to prevent the conversion of a precancerous cell into a tumor, mainly due to the fact that the early events triggering the tumoral fate and the commitment to a new cancerous lineage remain basically unknown [3]. This lack of knowledge is sadly illustrated by the cases of women carrying *BRCA1* or *BRCA2* mutations, whose only chance to reduce their probability of developing breast cancer is to undergo prophylactic tissue amputation [4]. It is clear that the most crucial point in the biological history of a cancer is the transition from a normal target cell to a cancerous one. However, the developmental mechanisms controlling the establishing of a tumoral cellular identity, which are essential for the cancer to arise in the first place, have received little attention. The main focus of both basic and translational research has been the altered controls of cellular proliferation in malignant cells. This has been reflected in the therapeutic approaches used to treat the patients: in general, most of the anti-cancerous treatments are directed against the mechanisms behind the abnormal proliferation of cancerous cells. However, these therapies are unspecific, with many side effects caused by their high toxicity and, in the end, unable of eradicating the disease in a large percentage of the cases. Therefore, an unmet need in cancer research is to understand how to neutralize the mechanism(s) that convert a normal cell into a cancerous one in the first place.

Douglas Hanahan and Robert Weinberg condensed the complex biology of cancer cells into nine hallmarks, “nine essential alterations in cell physiology that collectively dictate malignant growth” [5]. Cancer cells are the basis of the tumoral disease: they give rise to the tumors and drive the progression of the disease, carrying the oncogenic and tumor suppressor mutations that define cancer as a genetic disease [5]. In spite of their importance, we still do not fully understand the mechanisms that lead to their appearance, or at least we do not know enough so as to have a significant impact on cancer mortality [6]. As a consequence, our advances in cancer treatment are incremental and mainly empirical, with successive clinical trials leading to slightly better therapeutic options that, although they might provide some benefit, do not bring an end to the disease [7].

Therefore, an in-depth understanding of cancer requires a more detailed knowledge of the mechanisms triggering malignant growth, and it is essential if we want to identify the molecular culprits of cancer maintenance [3]. Despite this, all the aspects related to the deregulation of the normal developmental mechanisms that take place in tumorigenesis have received little attention when trying to define the main features of cancer. However, this is a key aspect since, if cellular fate could not be changed, cancer would be impossible, since only normal, non-pathological cell types would exist. Therefore, the mechanisms establishing and regulating cellular identity play an essential role in allowing the appearance of aberrant cancerous cell types; hopefully, the understanding of these mechanisms might be the key to the total elimination of cancer cells in the patients. In this review, we discuss the importance that oncogenes have in establishing the identity of the tumor cells, and how reprogrammed cells participate in the disease evolution. A deeper knowledge of this, so far largely neglected, mode of action of the oncogenes should help us to develop new ways to attack cancer.

## 2. Oncogene Addition versus Reprogramming Leukemia Predisposition

**Figure 1:**
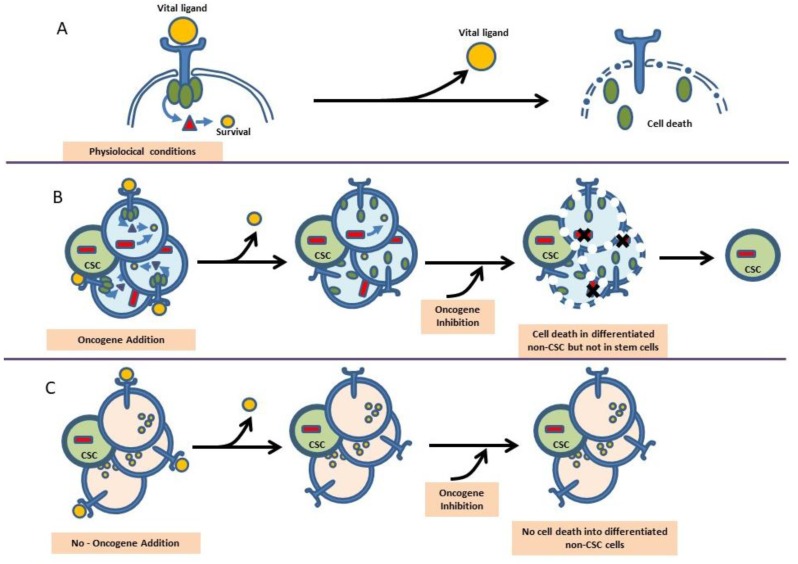
Oncogene addition versus reprogramming cancer predisposition. (**A**) In physiological conditions, the survival of a cell is determined by a specific survival pathway that needs to be activated by a vital ligand recognized by a specific receptor. If this vital ligand is removed from the niche/environment, the cell will die. (**B**) The oncogene addition hypothesis implies that a tumoral cell is able to grow independently of the survival signals because the oncogene activates survival pathways that maintain the tumor cell alive without vital ligands in the niche. The oncogene addition model implies that the inhibition of the oncogene in the tumor cells leads to the tumor cell death, as the survival pathways are no longer active. But under this scenario the evidence from the clinic indicates that only non-CSCs die. (**C**) The tumor reprogramming model of cancer initiation suggests that tumor stem cells are not oncogene addicted because there is a different function for oncogenes within CSCs. In this model, the target cancer cell of origin is not addicted neither to the oncogenes nor to the environmental signals.

Many years of research have shown that oncogene expression is necessary not only at the earliest stages of cancer development, but also for the posterior maintenance of the disease. Therefore, since the discovery that human cancers carry mutated oncogenes, these have been regarded as primary potential therapeutic targets. Indeed, in mouse models in which the expression of the oncogenes is driven by tissue-specific promoters, tumors arise frequently, but they regress when the oncogenic stimulus is switched off [8,9,10], suggesting that cancer cells are oncogene-addicted [11]. These findings seem to point to a homogeneous mode of action of oncogenes throughout tumor life, since the removal of the cancer-inducing oncogene will lead to tumor regression in these models (Figure 1). This model is fitting with the fact that, in human cancers, due to the clonal nature of the disease, all cancer cells carry the same initiating oncogenic lesions.

However, human cancers also present a very high degree of cellular heterogeneity [12], an indication that, in the oncogenic process, the nature and identity of the target cells suffering the action of the oncogene can be of great importance, especially since therapies based on the aforementioned current working model of cancer are incapable of eradicating cancer in humans [7]. On the contrary, the observations suggesting that the mode of action of oncogenes is not homogeneous throughout all the different cancer cell types would explain why anti-oncogene targeted therapies present different efficiencies against the different cellular stages of cancer evolution [7].

**Figure 2:**
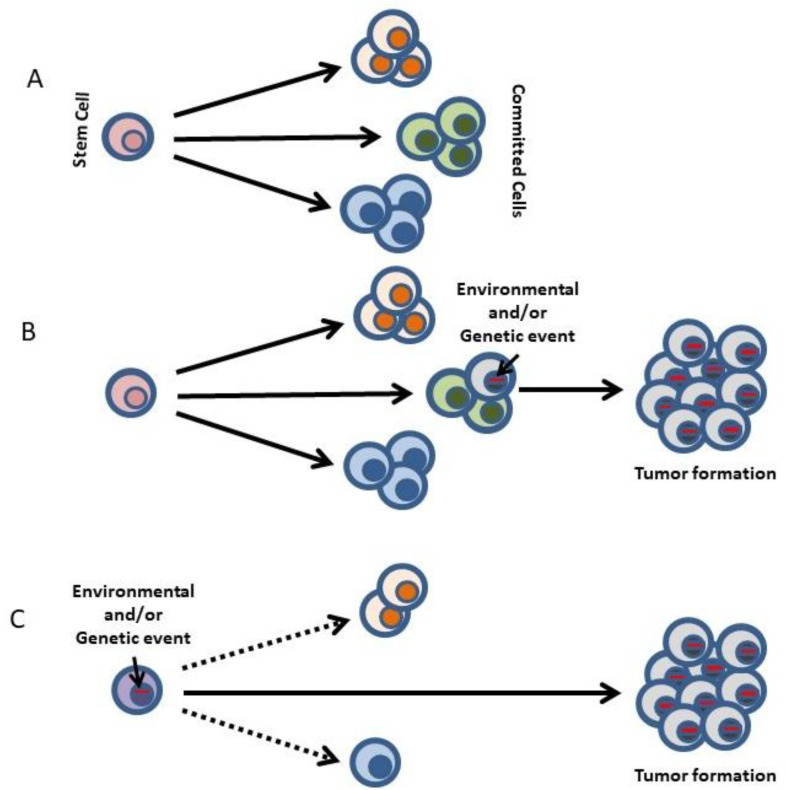
Proposed model for the role of human cancer gene defects in the making of a leukemia. (**A**) In the development of every normal tissue, a small pool of multipotent stem cells maintains multiple cell lineages. (**B**) Traditionally, human cancer genetic defects have been thought to act on cells already committed to a differentiation program. Hence, the cancer phenotype closely resembles that of the initial differentiated target cell. (**C**) Normal uncommitted stem and progenitor cells are the targets for transformation in some human cancers, and the human cancer-gene defects into these cells are the instigators of lineage choice decisions, which are therefore dictated by the oncogene and not by the cell-of-origin phenotype. Consistent with this is the finding that forced expression of these genes in stem cells can select or impose a specific cancer-lineage outcome. This explains why specific gene defects are usually found only in one type of cancer (see text for details). Dotted lines depict cell lineage choices that were not imposed by the oncogene.

This concept fits well with a model of cancer in which the tumor is generated and maintained in a hierarchical manner similar to that of the normal stem cell-driven tissues, like the hematopoietic system (Figure 2). In such a tissue, genetic programming of the stem cells is all what is required to give rise to all the differentiated cells forming the tissue, and the genetic information responsible for the stem cell programming does not need to be anymore present within those mature cells that form the tissue. When extrapolated to cancer formation, this concept would imply a potentially different role for the oncogenes at the level of the cancer stem cells (CSCs) [3,13]. Indeed, if cancer is generated by a malignant stem cell reprogramming process, the oncogenes initiating tumor formation might not be required for tumor progression [14,15].

This model also explains how, in the evolution of several human tumors, a pre-cancerous lesion can be stably maintained as the only proto-oncogenic alteration in an abnormal cell population that will only give rise to a full-blown tumor in response to secondary hits [16,17]. This initiating lesion is the driving force in the reprogramming process, essential for the acquisition of a tumoral phenotype. However, once its function in oncogenic reprogramming has been performed, this initiating hit would become just a passenger mutation within the CSC, having either no function or even performing a different one, unrelated to the initial reprogramming one, for example in tumor proliferation. This reprogramming model would explain why targeted therapies focused on the oncogenes can fail in eliminating all cancer cells, in spite of their initial efficacy against the cells composing the main tumor mass; a good example of this apparent paradox is imatinib, that fails to kill BCR-ABL^+^ CSCs because somehow it cannot block the reprogramming capacity of the fusion oncogene in the stem cell compartment [15,18,19].

In tumoral cells from human patients it is impossible to separately study the potential different roles of the oncogenes at the different stages of tumor development, due to the advanced stage of the tumors at diagnosis and the accumulation and superposition of numerous driver and passenger mutations. Indeed, in order to reveal the existence of a lack of homogeneity in the action of oncogenes throughout the biological history of the tumor, we need a system that allows us to isolate the function that the oncogene is playing in the first steps of cancer, in the cancer cell-of-origin. Ideally, one would need a system in which the oncogene expression was restricted to the stem/progenitor compartment in order to demonstrate that the posterior expression of the oncogene (as happens in human tumors) is in fact not necessary for tumor progression once the tumoral reprogramming has already taken place, and is therefore dispensable for cancer progression or maintenance (Figure 2).

**Figure 3:**
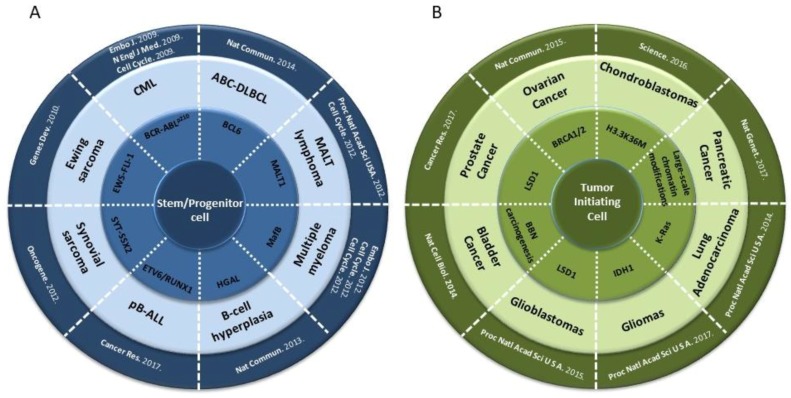
Driving of cancer by a malignant epigenetic stem cell rewiring. Different types of mesenchymal (**A**) and epithelial (**B**) malignancies have shown that the contribution of oncogenes to cancer development is mainly mediated through the epigenetic reprogramming of the cancer-initiating target cell. As illustrated, specific genotype alterations associated to human cancer (medium circle) give rise to specific phenotypes (outer circle) when targeted to the stem cell/progenitor compartment (see text for details). Chronic Myeloid Leukemia (CML), Activated B-Cell Diffuse Large B-Cell Lymphoma (ABC-DLBCL), precursor B-cell Acute Lymphoblastic Leukemia (pB-ALL), *N*-butyl-*N*-4-hydroxybutyl nitrosamine (BBN).

This conceptual framework has recently given rise to experimental settings in which different oncogenic lesions, each linked to a specific type of hematopoietic cancers, have been targeted to the hematopoietic stem/progenitor cellular compartment of genetically engineered mice by using the locus control region of the *Sca1* (“Stem cell antigen-1”) gene. It is a mouse glycosyl phosphatidylinositol-anchored cell surface protein (GPI-AP) of the *LY6* gene family. It is the common biological marker used to identify hematopoietic stem cell (HSC) along with other markers and plays a role in hematopoietic progenitor/stem cell lineage fate. In this setting, it has been shown that the different lesions can epigenetically re-program the targeted stem cells and create a differentiation state from which tumor cells with different properties emerge heterogeneously [15,20,21,22,23,24] (Figure 3). Overall, this new view on oncogenesis not only reveals a novel function for oncogenes in cancer, but also provides evidence for a previously unconsidered model of tumorigenesis, in which the programming of the cancerous cellular identity has already occurred at the level of stem/uncommitted cells, therefore showing a role for oncogenes in the timing of cancer initiation.

## 3. Restriction of Lineage Options during the Making of Leukemia

A conceptually clarifying example of the power of the aforementioned experimental setting to recapitulate the characteristics of human cancers is chronic myelogenous leukemia (CML), a widely accepted stem cell disorder characterized by the presence of the chimeric *BCR-ABLp210* oncogene. When the expression of BCR-ABL is restricted to the Sca1^+^ cells in mice, the animals develop CML [15]. This model is designed so that the oncogene expression is switched off in the differentiated cells that form the main mass of the tumor, although leukemia initiation has taken place within the stem cell/progenitor population. The fact that CML arises in mice under these circumstances indicates that the absence of BCR-ABL expression is not required for the generation of differentiated tumor cells (Figure 2). These results connect tumorigenesis with the reprogramming of early progenitors and strongly support the existence of a reprogramming-like mechanism in cancer development.

CML is a paradigmatic stem-cell-driven cancer in humans, but we reasoned that, assuming the tumoral reprogramming theory as we have previously mentioned, a similar experimental approach as the one used to model *BCR-ABLp210^+^* CML could also be used to reproduce in the mouse the genotype-phenotype correlation (specific oncogene/specific tumor) found in other human cancers. A challenging system to test this hypothesis would be a tumor whose main cell type is a mature differentiated cell, like in the case of multiple myeloma (MM) or mature B-cell lymphoma. In fact, it has been shown that both MM (induced by the *MafB* oncogene) and B-cell lymphoma (induced either by the *MALT1* or the *BCL6* oncogenes) phenotypes and biology can be accurately mimicked in mice with the same *Sca1*-mediated stem cell targeting system described before [20,21,22,23] (Figure 3). These results implicated for the first time the stem cells in the pathogenesis of MM and B-cell lymphoma. Also, the fact that both hematopoietic tumors can be generated in mice by limiting oncogene expression to hematopoietic stem/progenitor cells (HS/PCs) implies that eliminating oncogene function beyond the stem cell stage does not interfere with the generation of later tumoral developmental cell types, and suggest that the oncogene is programming in the stem cells an epigenetic program that in some way persists during differentiation and will finally lead to a mature tumoral phenotype of MM or B-cell lymphoma [21,22,23,25,26]. Therefore, we postulate that cancer-initiating oncogenes epigenetically modify target genes that remain in this “poised” state in the mature tumor even when the oncogene is not present anymore (Figure 2).

## 4. Epigenetic Reprogramming in Non-Hematopoietic Tumors

Other examples of tumoral stem cell reprogramming, in which the induction of a new tumoral fate by the oncogene takes place at the stem cell level (as opposed to reprogramming to pluripotency, which is initiated from a differentiated cell) have been described for other types of non-hematopoietic tumors. For example, the *EWS-FLI-1* fusion gene, associated with most Ewing sarcoma tumors, triggers the expression of the embryonic stem cell genes *OCT4*, *SOX2*, and *NANOG* when present in human pediatric mesenchymal stem cells but not in adult ones, and it reprograms them to give rise to Ewing sarcoma cancer stem cells [27]. Similarly, the synovial sarcoma-associated oncogene SYT-SSX2 can reprogram mesenchymal stem cells by promoting their differentiation towards a pro-neural lineage, in what most likely constitutes the primary tumorigenic event in this type of cancer [28]. A similar scenario has recently been described in the genesis of chondroblastomas [29].

This driving of cancer by a malignant epigenetic stem cell rewiring is, however, not exclusive to mesenchymal-derived cancers, but rather represents a common tumoral mechanism that is also at work in epithelial tumors like lung carcinomas [30], bladder cancer [31], skin carcinomas [32], ovarian carcinomas [33], pancreatic carcinomas [34], brain tumors [35,36], and prostate carcinomas [37] (Figure 3).

These results prove that, when oncogenic proteins are expressed in stem or progenitor cells, they can have a highly selective impact in differentiation. This, in turn, helps explaining the strikingly consistent associations between each given chromosomal translocation, its resulting chimeric oncogene and the final phenotype of the cancer it triggers. Altogether, the evidence supports a new vision of cancer mainly as a disease of cellular differentiation, much more than just a proliferative disorder, and asks for a reconsideration of the function of oncogenes. We should also insist on the fact that this ‘hit-and-run’ reprogramming model for oncogene activity is not something happening only in pathological conditions. Indeed, during normal hematopoietic development, for example, molecular cues such as IL7 and erythropoietin are required to trigger specific differentiation programs but are not required once the programs have been established.

This new conceptual framework, supported by the experimental findings and by the frequent therapeutic failures in cancer human patients, also has important implications in the clinical management of cancer. Certainly, if cancers develop through a reprogramming mechanism, then the oncogenes, although necessary to initiate the tumor, might be dispensable for posterior tumor survival and/or progression. Oncogenes would then have a driving role in the reprogramming process, but be only passenger mutations afterwards, or have a secondary, unrelated role in more evolved tumor cell clones. For example, in human CLL, the susceptibility to generate malignant B cells is already present at the hematopoietic stem cell (HSC) stage, long before the cells become B cells [38]; consequently, patient-derived HSCs show an abnormal expression of lymphoid-related genes, reflecting their cell-autonomous aberrant priming into the B-cell lineage. These findings, therefore, have important implications for the therapeutic targeting of tumoral cells.

For example, in the case of CML, with one of the most commercially successful oncogene-targeted therapies so far, the data from *Sca1-BCR-ABLp210* animal models showed that the survival of CML stem cells was independent on the kinase activity of BCR-ABL, therefore indicating that curative approaches in CML should most probably focus on kinase-independent mechanisms of resistance [15]. These results on the failure of imatinib to eradicate CML in *Sca1-BCR-ABLp210* mice have been later confirmed in human patients [39,40,41,42,43], and this is a clear example of how a good preclinical model can anticipate the human CSC-therapeutic response. These results on the role of *BCR-ABLp210* in CML development show that leukemia stem cells might not be oncogene-addicted (Figure 1), and are most likely relevant to many other cancers (multiple myeloma, MALT lymphoma, CLL, etc.). Furthermore, these findings challenge the current accepted/working model of the role of oncogenes and support the hypothesis that mature hematological malignancies may be initiated by an inappropriate lineage-decision making process at the HSC level.

## 5. Hematopoiesis and Leukemia Are Both Lineage Decision-Making Processes

The most important functional characteristics of HSCs are their capacity for self-renewal and their multilineage differentiation potential. Traditionally, the generation of differentiated cells from HSCs was thought to occur through a series of dichotomic branching steps diverting into mutually exclusive stable progenitor states. However, recent work has shown that hematopoiesis occurs through a mechanism of continuous lineage priming [44] and therefore the architecture of the system is much less compartmentalized than previously considered, and also more versatile in terms of lineage plasticity, since developing progenitors can use different unusual pathways and/or have hidden potentials, so that it could very well be that “defined” progenitor populations are in fact mixtures of cells with several differentiation capabilities. This vision also changes our idea of cellular commitment, if developing hematopoietic cells are in fact being gradually biased towards a certain fate without sudden black-or-white developmental steps. A very important element of this vision is that these progressive developmental biases are physiological rules that can be bent or even broken by both intrinsic and extracellular factors in pathological conditions, a fact that has clear implications to the understanding of the origins and progression of malignant transformation in a setting in which, as we have stated, cancer would mainly be a disease of cellular differentiation (Figure 2).

**Figure 4:**
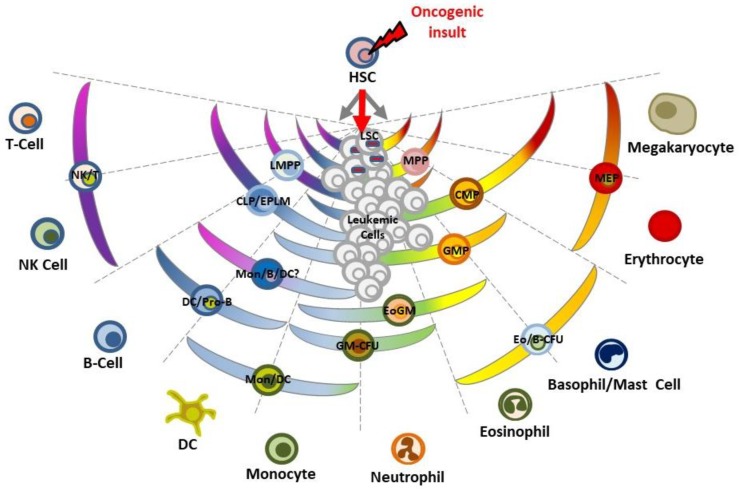
Schematic representation of the emergence of LSCs in the making of a leukemia. A mutation occurs in HSCs leading to the emergence of aberrant pre-leukemic HSCs. These aberrant pre-leukemic HSCs self-renew and expand within the HSC compartment. Pre-leukemic HSCs give rise to a high number of lineage-committed progenitors harboring this identical mutation. This leads to an increased chance of acquiring the additional oncogenic/environmental events, which finally transform the aberrant progenitor cells from pre-leukemic HSCs into the leukemic stem cells (LSCs). Loss of differentiation potentials is essential for the emergence of LSCs. LSCs are reprogrammed by an oncogenic insult to an invariant cell lineage. Hematopoietic stem cell (HSC), Multipotent progenitor cell (MPP), Lymphoid-primed MPP compartment (LMPP), Common myeloid progenitor (CMP), Common lymphoid progenitor (CLP), Early progenitor with lymphoid and myeloid potential (EPLM), Granulocyte-macrophage progenitor (GMP), Monocytes (Mon), Eosinophil-granulocyte-macrophage (EoGM), Granulocyte-macrophage progenitor-colony forming unit (GM-CFU), Dendritic cell (DC), Megakaryocyte-erythroid progenitor (MEP), Natural Killer cell (NK), Eosinophil/Basophil-colony forming unit (Eo/B-CFU).

This new point of view deepens our understanding of the biology of leukemia and its origins. In several types of leukemia it has been shown that the pre-leukemic stem cell (pre-LSC) possesses multilineage potential; this is the case in CML, where pre-leukemic *BCR-ABLp210^+^* stem cells can give rise to all different blood cell types (Figure 4). This is also the case for CMLs associated with a mutant *RAS* allele, in which this mutant *RAS* can be found in all mature lineages [45]. In myelodysplastic syndromes, a multipotent malignant stem cell is behind the development of refractory anemia (RA), RA with ringed sideroblasts or RA with excess blasts [46]. In all these cases, the existence of a pre-LSC with multilineage differentiation potential suggests that initiating mutations arise in a normal HSC and that afterwards, through the acquisition of additional mutations triggered by secondary events, the initiated clone will evolve to produce a sub-clone of lineage-restricted malignant blasts (Figure 4). This two-hit model of leukemogenesis relies on the stepwise acquisition and collaboration between two main groups of mutations: (i) those affecting genes of transcriptional or epigenetic regulators that can modify or restrict lineage options (e.g., generation of chimeric oncogenes such as *RUNX1-RUNX1T1* or *PML-RARA*, *BCR-ABLp190, ETV6-RUNX1* or mutations in *CEBPA*, *PAX5* or *NPM1*) and, (ii) those activating signal-transduction pathways that confer survival or proliferative advantages (e.g., mutations in *FLT3*, *RAS* or *KIT*). Therefore the pre-leukemic oncogenic lesion is stably maintained as a single alteration in an abnormal cell population, but will only progress to an open leukemia when secondary hits occur [16,17]. Therefore, although the cells that suffer the initiating leukemic hit possess multi-lineage potential, LSCs are reprogrammed by this oncogenic hit and their lineage decision-making becomes restricted or biased.

It seems therefore that tumoral reprogramming and aberrant lineage-programming are crucial characteristics at the root of cancers including leukemia. The neural stem cells from malignant glioblastoma can be reprogrammed to induced-pluripotent stem cells (iPSC). These iPSC can then be differentiated into mesodermal lineages, and along this developmental pathway they lose their malignant nature, but they maintain it when they are differentiated into neural cells [47]. Similarly, primary human Philadelphia chromosome-positive B cell acute lymphoblastic leukemia (B-ALL) cells can be reprogrammed into non-leukemic macrophages, overriding the malignant differentiation block into the B cell lineage [48]. These findings underscore the fact that the cancerous condition is in some way linked to the fact that the cells have been programmed to adopt a specific given lineage. Then the key questions are: Which is the normal developmental stage that is being programmed? and at what stage does this occur within the leukemic development itself?

The leukemic conversion is only possible if the normal cell that gives rise to the leukemia, the leukemia cell-of-origin (LOC), has the necessary developmental plasticity to tolerate the reprogramming and react to it by changing its fate. On the other hand, the oncogenic event(s) triggering LOC malignant conversion cancer must also have a reprogramming capacity to be able to promote such a change in cellular identity [49].

It is generally accepted that tumoral progression is a multi-hit process and, also from a tumoral reprogramming perspective, the different aspects of normal cellular biology must be progressively altered to finally give rise to a full-blown tumor [5]. Under normal conditions, HS/PCs are slowly moving towards lineage biases, diversifying and differentiating towards their final cellular identities. The requirement of multiple hits for full tumor development is in relationship to the fact that the changes required to revert or deviate cells from their normal non-pathogenic fate are inherently disfavored developmentally, and biological barriers are in place to ensure that cells do not easily change their identity in order to minimize the risk of malignant transformation.

This biological reluctance of the cells to being reprogrammed by an oncogene to a tumor phenotype is illustrated by recent studies on stem-cell-based animal models of human cancer. The loss of the p53 tumor suppressor is a frequent occurrence in malignancy, and it has a clear function in facilitating pathological reprogramming to a malignant phenotype. In a stem-cell-based transgenic model of multiple myeloma, the loss of p53 accelerates the appearance of the disease by allowing the *MafB* oncogene to drive a much more efficient malignant transformation [22,25]. Something similar happens in the case of mucose-associated lymphoid tissue (MALT) lymphoma driven by the *MALT1* oncogene [21]. In a stem-cell-based model of CML [50], the restoration of p53 activity in already established cancers slowed the progression of the disease and prolonged the survival of diseased animals by causing the apoptotic death of leukemic progenitors, one more demonstrating the importance of reprogramming in lineage decision-making towards a tumoral fate.

## 6. The Importance of Environmental Signals in the Making of Leukemia

We have seen that precancerous lesions can exist as stable single alterations maintained in an abnormal, but not cancerous, cell population that will only progress to full-blown cancer as a result of secondary hits [16,17]. One of the best examples of this is childhood B-ALL, in which the first oncogenic hit, through a stem cell tumoral epigenetic reprogramming mechanism, gives rise to a preleukemic clone that remains harmless until its carrier is exposed to common infections [24,51,52]. This infection exposure would never cause leukemic development in healthy individuals (i.e., persons not carrying a preleukemic clone). Also, human epidemiological studies show a positive association between body weight at birth and the risk of developing childhood leukemia. This implies that, although some epigenetic reprogramming can be observed immediately after exposure to exogenous or endogenous agents, both aberrant epigenetic programming and altered disease susceptibility may manifest only later in life, long after the exposure took place. However, there are not known differences in epigenetic reprogramming between childhood and adults with leukemia. Together, these data lead us to propose that leukemia as a result of epigenetic reprogramming is a type of gene–environment interaction that can cooperate with a genetic predisposition, not by inducing mutations, but by reprogramming the epigenome to modulate gene expression in order to promote leukemia development. By dissecting how epigenetic reprogramming increases leukemia risk, we may not only be able to better identify who has an increased risk of developing leukemia from early life environmental exposures, but may also be able of developing interventions that can reverse the epigenetic effects of the tumor epigenetic reprogramming to decrease leukemia risk associated with this type of gene–environment interaction.

## 7. Therapeutic Intervention in Leukemia and the Prospect of Modifying the Making of Leukemia

The understanding of cancer as a LSC-dependent aberrant tissue has deep implications for cancer treatment. Obviously, under the LSC conceptual framework, the LSCs should the primary targets of anti-cancerous therapy. However, since LSCs share most of their basic biological properties with normal, non-pathologic stem cells, therapies directed against LSC pathways might also unintentionally eliminate normal resident stem cells.

**Figure 5:**
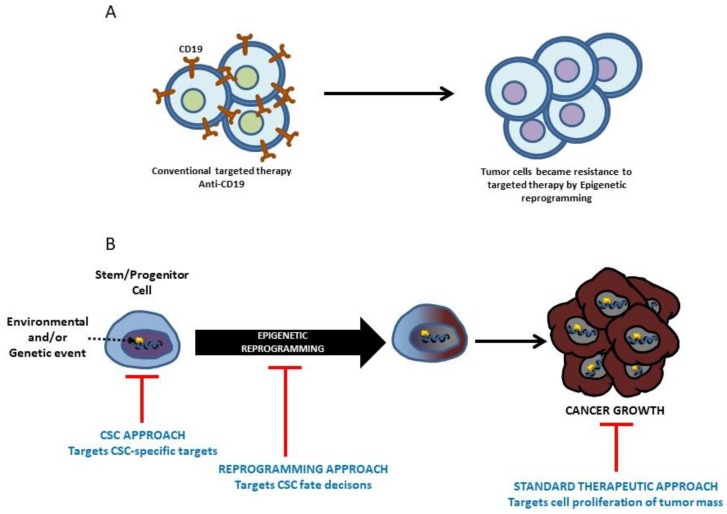
Epigenetic reprogramming and cancer therapy. (**A**) Epigenetic reprogramming can be the mechanism used by tumors to evade CD19 CAR immune therapy. (**B**) Epigenetic reprogramming can be exploited in therapy to kill leukemia/cancer stem cells. Recent findings indicate that rewiring the epigenetic programming of tumor cells is a viable prospect (see text for details).

We have seen how the main contribution of oncogenes to tumor development is not their proliferation-inducing capability, but rather their capacity for reprogramming the LSC epigenome. This capacity of making leukemia in such a way that the maintenance of oncogene expression is not required for the posterior generation of differentiated tumoral cells seems to be a common mechanism of determination of cancerous identity and, as such, it should change our understanding of how the “hallmarks of cancer” are acquired during tumor development. In this sense, it has recently been shown that epigenetic reprogramming can be the driving force behind intra-tumoral heterogeneity [53], and can also be the mechanism used by tumors to evade CD19 CAR immune therapy [54,55] (Figure 5).

With the new animal models generated within this stem cell reprogramming paradigm, we can now study how different cancerous stages develop from the very beginning, and we could unlock the potential to provide great advances in human cancer medicine. Since assessing the effects of therapies on the rare LSCs that are the responsible for relapse is almost impossible in the patients, the development of these treatments will have to heavily rely on the use of accurate preclinical models and preclinical assays.

The key factor in this new view of cancer specification is the setting of a new regulatory circuitry by epigenetic reprogramming. This opens a new door for therapeutic opportunities since in these last tears we are learning more and more about how to genetically or pharmacologically manipulate the epigenetic status of cells. In fact, epigenetic therapeutic protocols have already been incorporated in some cases to standard chemotherapy regimens as a potential improvement in the treatment of, for example, relapsed pediatric acute lymphoblastic leukemia [56].

Also, in more experimental settings, cancer cells have been reprogrammed to non-tumoral fates, losing their malignancy. For example, it is possible to produce even mouse embryos from brain-tumor-derived cells [57] and to reprogram embryonal carcinomas [58] or melanoma cells by using nuclear transplantation [59]. Similarly, B-ALL cells have been reprogrammed to an alternative lineage cell fate without a malignant phenotype [56]. Also, it has recently been shown that epigenetic reprogramming can be exploited in therapy to kill leukemia stem cells [60] and can also be used to treat pediatric brain cancer [61,62]. These findings support the underlying theory and indicate that rewiring the epigenetic programming of tumor cells is a viable prospect. Also, it is not unreasonable to expect that LSCs from different cancer types will share many similarities, so that similar LSC-based therapeutic approaches could be successfully employed against different cancer types (Figure 5). In any case, like for any other type of therapy, a detailed understanding of the epigenetic rewiring is a prerequisite for any potential intervention.

## 8. Future Opportunities and Challenges

The new perspective of leukemia that is arising from the most recent results from advanced animal models is leading us to a better understanding of the biology of the disease and, at the same time, is forcing us to question long-standing beliefs about the role of oncogenes in leukemia generation.

We have seen that the exposure of plastic stem cells to the epigenetic reprogramming capacity of some oncogenes works as a new type of gene–target cell interaction in which oncogene exposure poises the epigenome to induce leukemia development [20,21,22,23,24]. In this model of action, oncogene-activating mutations would have a driving role in the reprogramming process at the leukemia cell-of-origin, but may become passenger alterations (or have a different, secondary role) at later stages. To increase the complexity of the problem, the phenotypic consequences of the epigenetic reprogramming can remain silent until triggered by later exposures (genetic and/or environmental) [24,51,63,64,65]. Of great importance is the fact that the setting of the epigenetic circuits that lead to tumor cell development is unidirectional. This implies that even brief exposure to an environmental agent can disrupt the normal epigenetic developmental programs and alter the epigenome for life [66]. Now we have the ability to model tumor stem cell generation in vivo for different types of cancer, with their respective inducing oncogenes. This opens up new possibilities for studying how the different cancer stages develop from the start. If we can understand how the oncogene–target cell interaction is regulated, then we might learn how to manipulate tumoral cellular identities and stages experimentally, a knowledge that could lead to tremendous advances in human cancer medicine.

Looking into the future, we are faced with the paradox that we still do not understand how the balance is regulated between cell intrinsic and environmental agents in developmental processes. Cellular pluripotency is a pre-requisite for the versatility of the organisms, giving them the capacity of evolving different types of specialized cells to face different challenges. However, pluripotency is a force that needs to be tamed in order to give rise to an adult organism composed of highly differentiated (and not therefore pluripotent) cells. In the last decade, we have learnt that the architecture of adult tissues is much more versatile and plastic than previously thought, and this is particularly important, for example, when responding to the demand triggered by infectious agents as to the specialized cells required to fight them.

New findings open new questions. For example, is the decision to initiate leukemia made at one single time point during the tumoral differentiation process, or is composed by a series of consecutive decisions required to switch to a leukemia cell fate? What is the precise nature of the epigenetic pathway triggered by the leukemia-initiating gene defect(s)? Last but not least, these findings on the mechanisms of cellular commitment to a tumoral fate are relevant not only to the understanding of the stem cell properties of leukemia and the development of new therapies, but also to regenerative medicine, since in this context it will be essential to have full control over the potential malignancy of reprogrammed cells.
